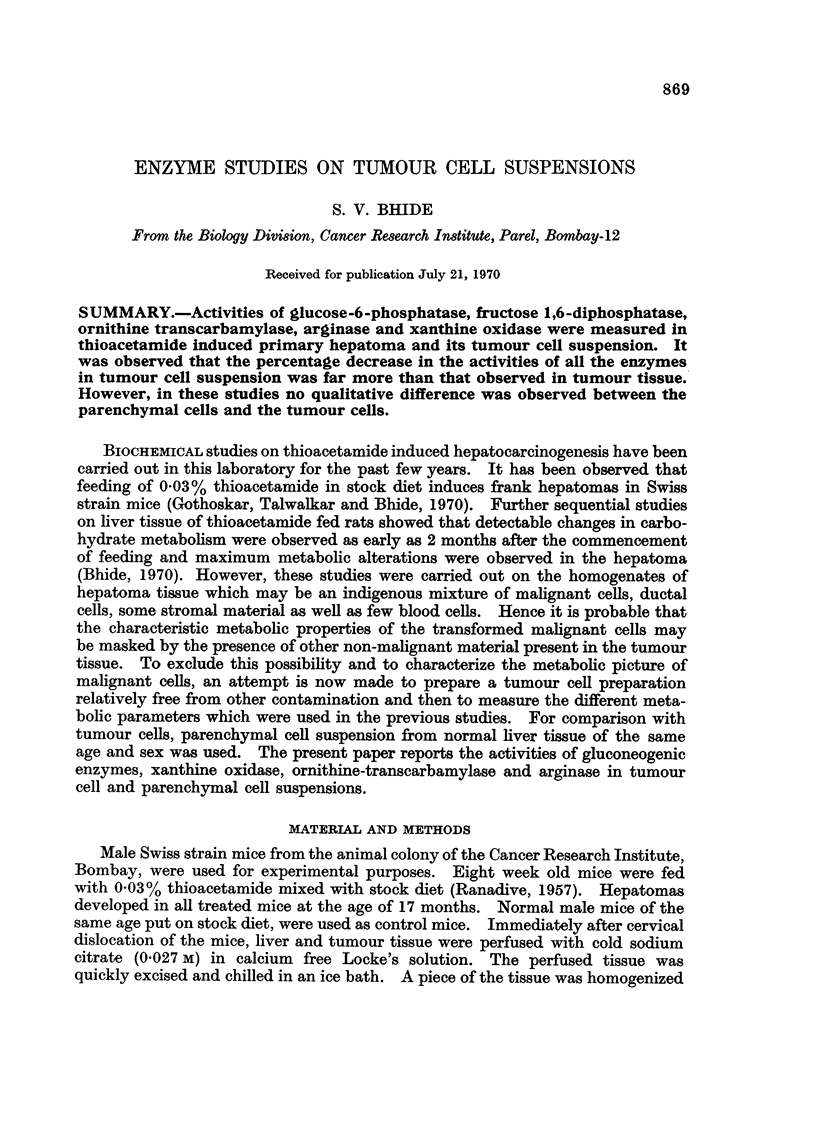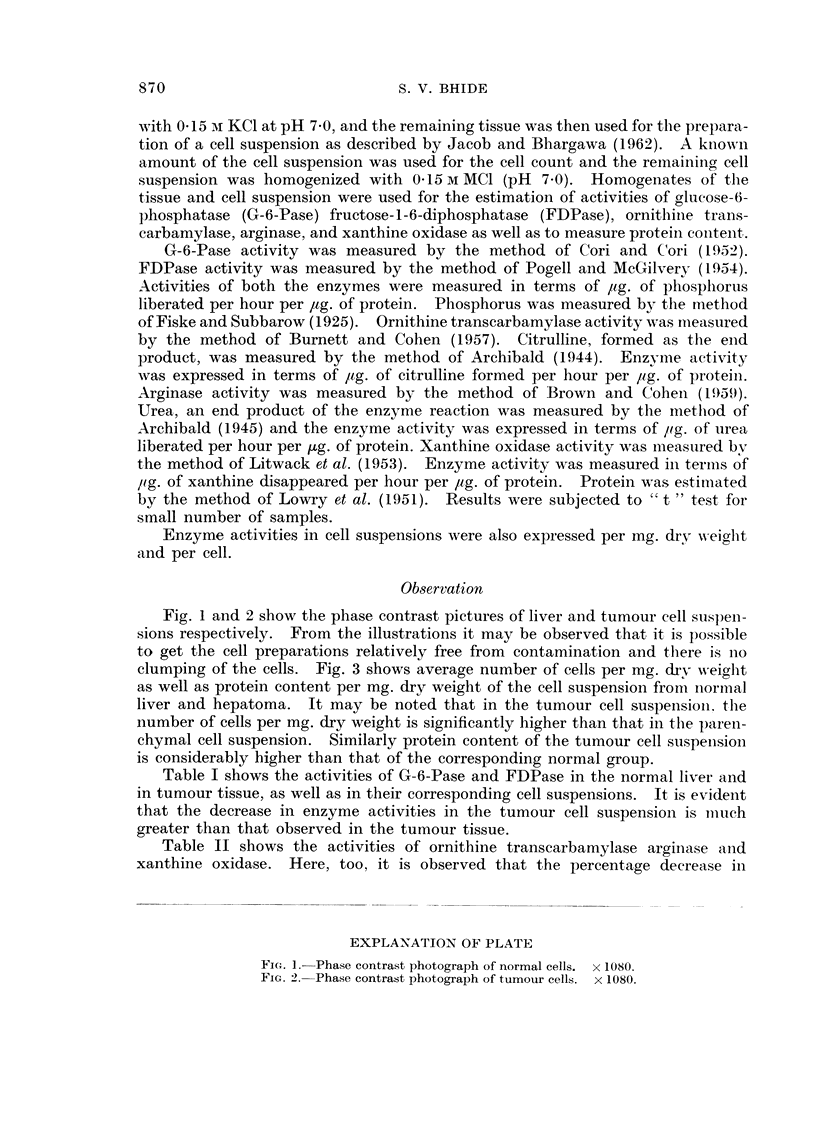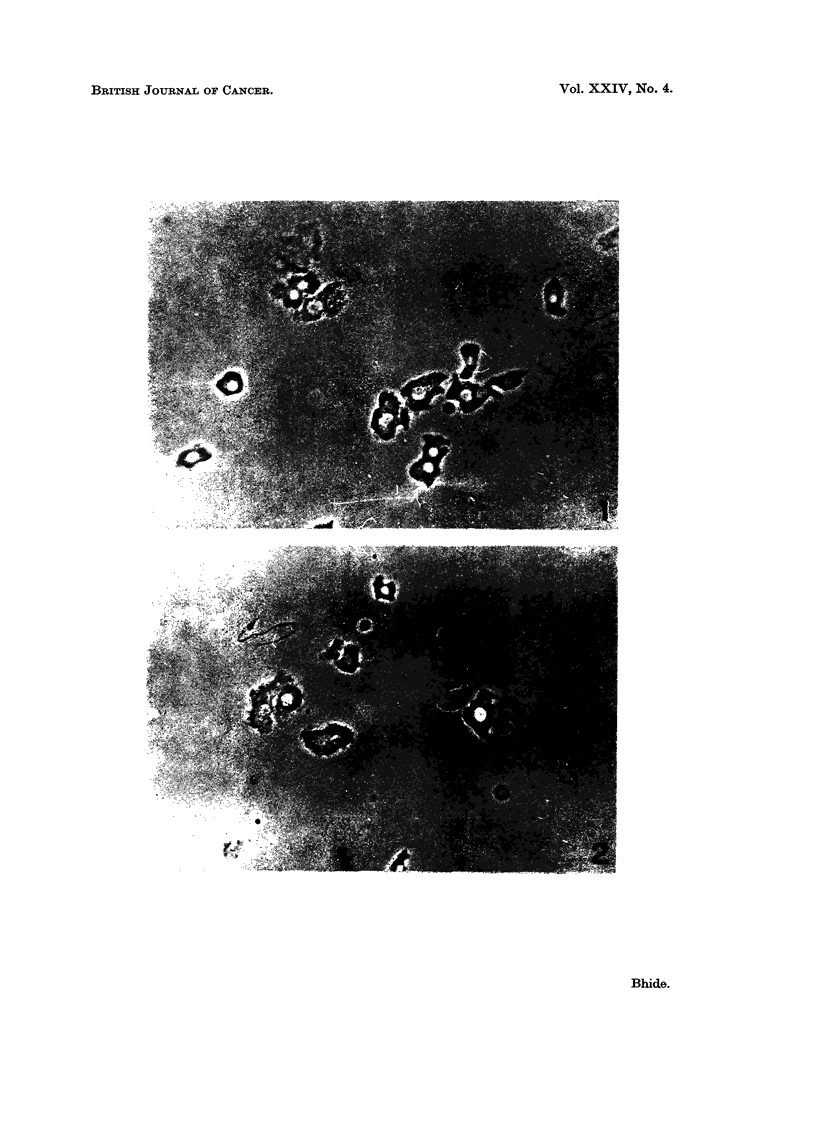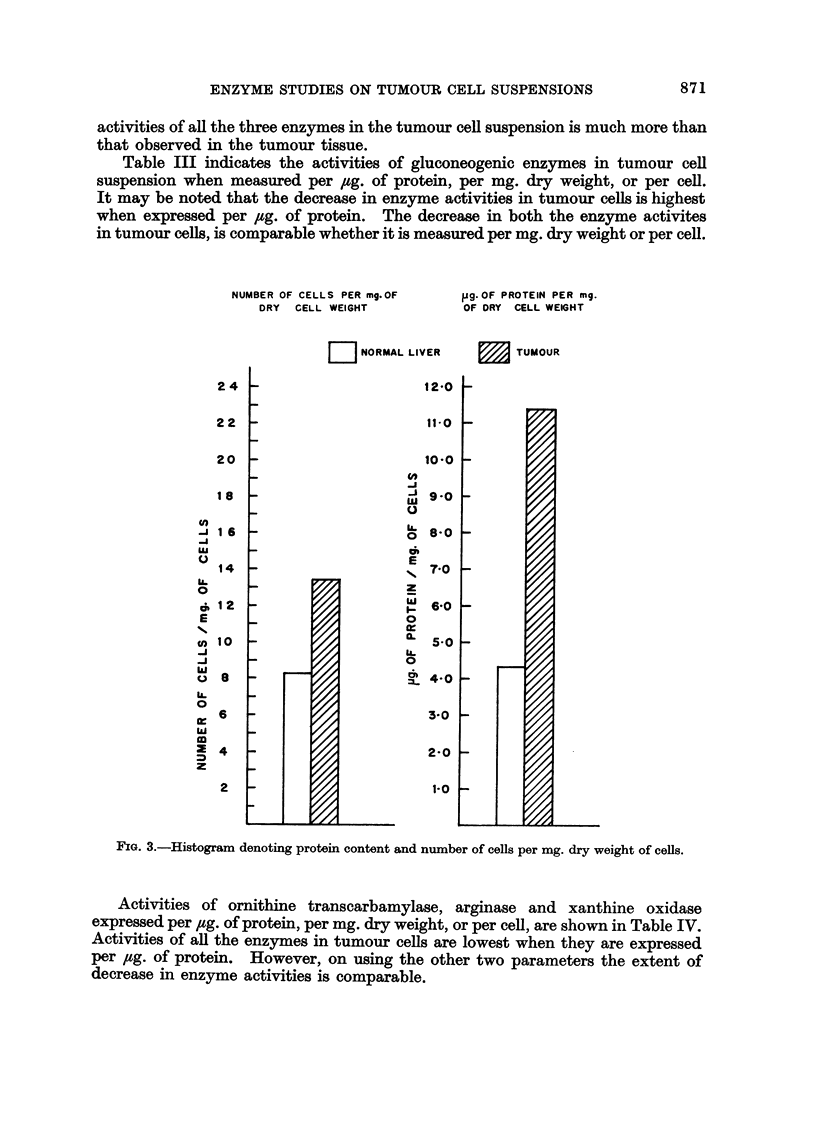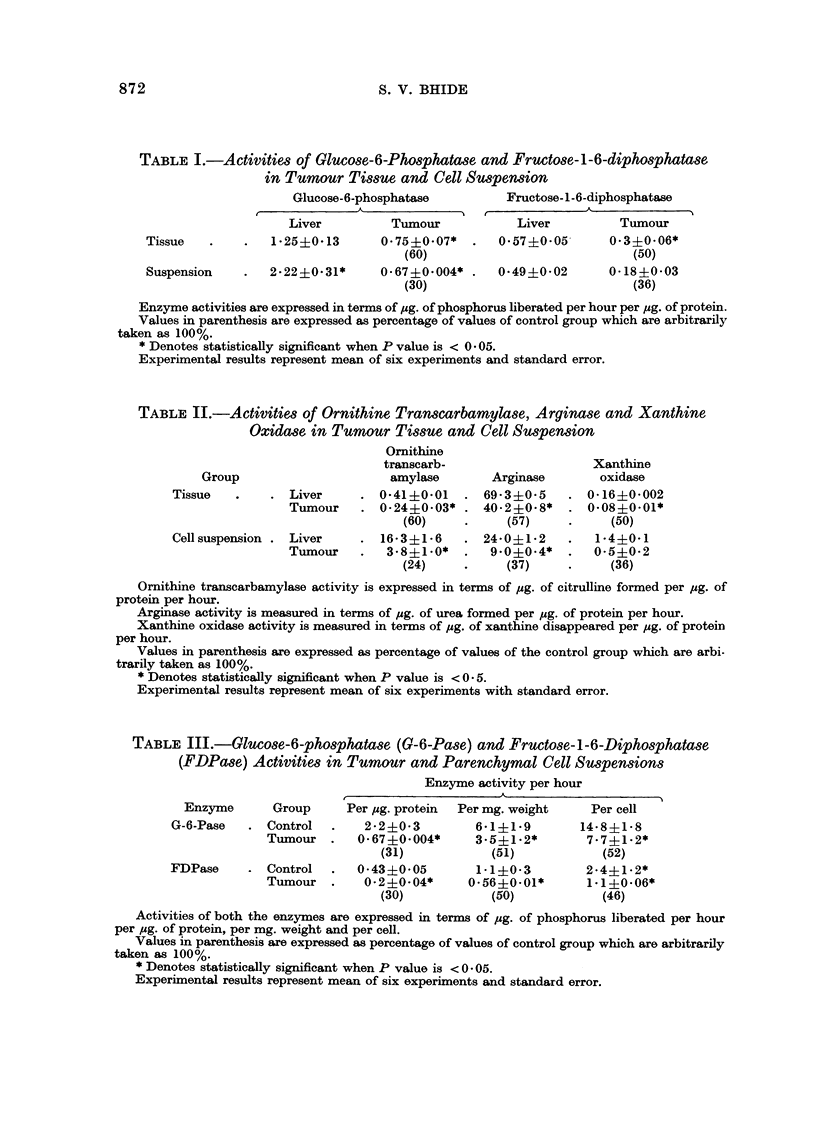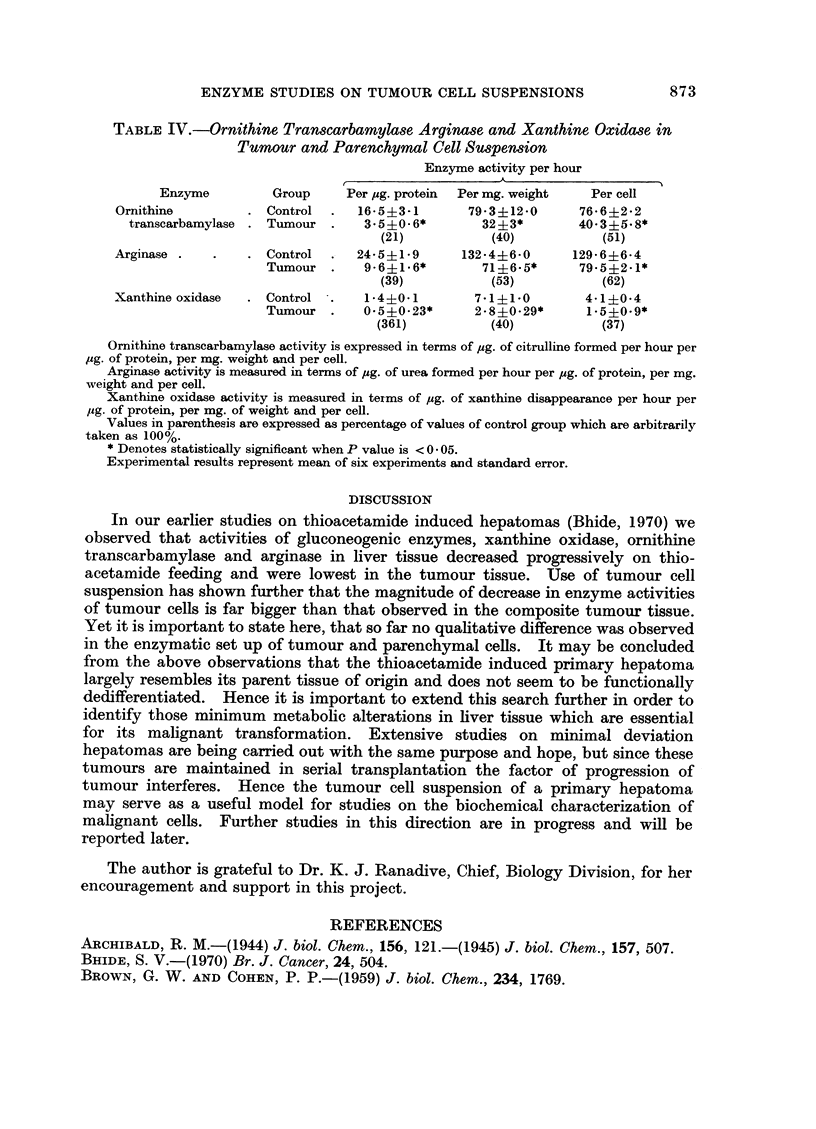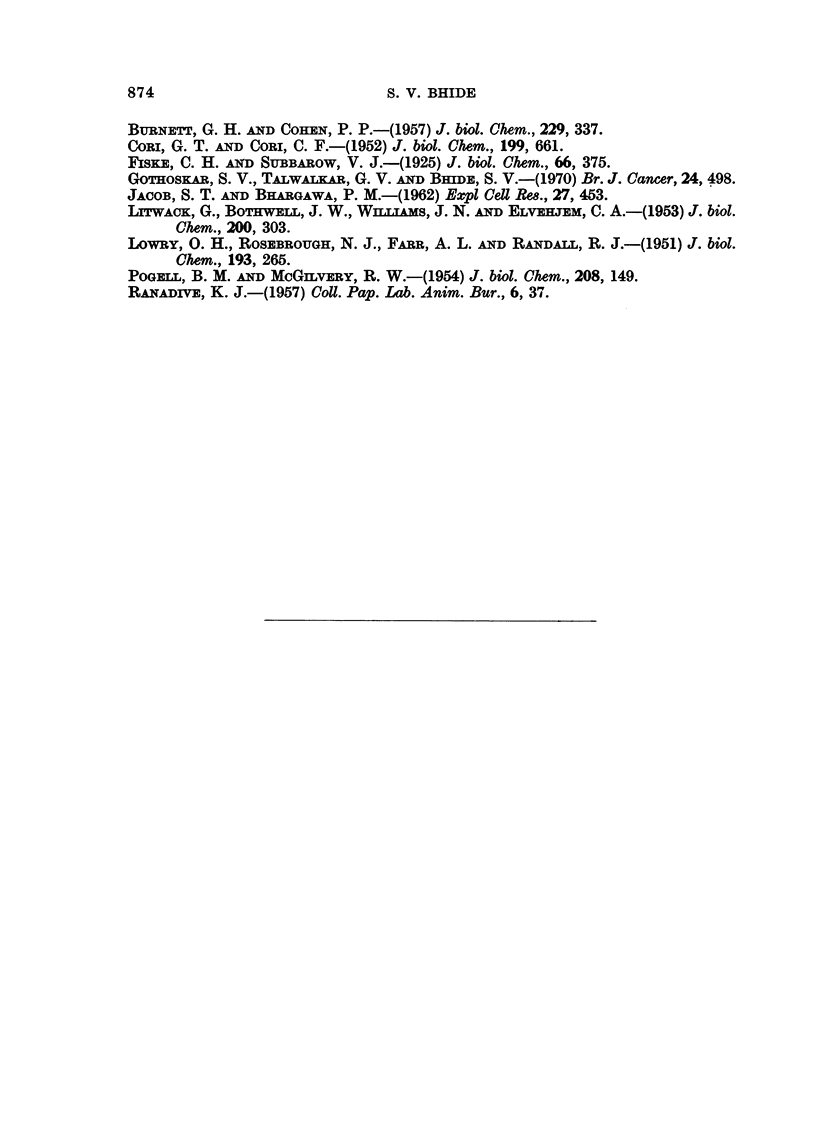# Enzyme Studies on Tumour Cell Suspensions

**DOI:** 10.1038/bjc.1970.103

**Published:** 1970-12

**Authors:** S. V. Bhide

## Abstract

**Images:**


### ENZYME STUDIES ON TUMOUR CELL SUSPENSIONS

S. V. BHIDE

*From the Biology Division, Cancer Research Institute, Parel, Bombay-12*

Received for publication July 21, 1970

**SUMMARY.—Activities of glucose-6-phosphatase, fructose 1,6-diphosphatase, ornithine transcarbamylase, arginase and xanthine oxidase were measured in thioacetamide induced primary hepatoma and its tumour cell suspension. It was observed that the percentage decrease in the activities of all the enzymes in tumour cell suspension was far more than that observed in tumour tissue. However, in these studies no qualitative difference was observed between the parenchymal cells and the tumour cells.**

BIOCHEMICAL studies on thioacetamide induced hepatocarcinogenesis have been carried out in this laboratory for the past few years. It has been observed that feeding of 0·03% thioacetamide in stock diet induces frank hepatomas in Swiss strain mice (Gothoskar, Talwalkar and Bhide, 1970). Further sequential studies on liver tissue of thioacetamide fed rats showed that detectable changes in carbohydrate metabolism were observed as early as 2 months after the commencement of feeding and maximum metabolic alterations were observed in the hepatoma (Bhide, 1970). However, these studies were carried out on the homogenates of hepatoma tissue which may be an indigenous mixture of malignant cells, ductal cells, some stromal material as well as few blood cells. Hence it is probable that the characteristic metabolic properties of the transformed malignant cells may be masked by the presence of other non-malignant material present in the tumour tissue. To exclude this possibility and to characterize the metabolic picture of malignant cells, an attempt is now made to prepare a tumour cell preparation relatively free from other contamination and then to measure the different metabolic parameters which were used in the previous studies. For comparison with tumour cells, parenchymal cell suspension from normal liver tissue of the same age and sex was used. The present paper reports the activities of gluconeogenic enzymes, xanthine oxidase, ornithine-transcarbamylase and arginase in tumour cell and parenchymal cell suspensions.

### MATERIAL AND METHODS

Male Swiss strain mice from the animal colony of the Cancer Research Institute, Bombay, were used for experimental purposes. Eight week old mice were fed with 0·03% thioacetamide mixed with stock diet (Ranadive, 1957). Hepatomas developed in all treated mice at the age of 17 months. Normal male mice of the same age put on stock diet, were used as control mice. Immediately after cervical dislocation of the mice, liver and tumour tissue were perfused with cold sodium citrate (0·027 M) in calcium free Locke's solution. The perfused tissue was quickly excised and chilled in an ice bath. A piece of the tissue was homogenized with 0·15 M KCl at pH 7·0, and the remaining tissue was then used for the preparation of a cell suspension as described by Jacob and Bhargawa (1962). A known amount of the cell suspension was used for the cell count and the remaining cell suspension was homogenized with 0·15 M MCl (pH 7·0). Homogenates of the tissue and cell suspension were used for the estimation of activities of glucose-6-phosphatase (G-6-Pase) fructose-1-6-diphosphatase (FDPase), ornithine transcarbamylase, arginase, and xanthine oxidase as well as to measure protein content.

G-6-Pase activity was measured by the method of Cori and Cori (1952). FDPase activity was measured by the method of Pogell and McGilvery (1954). Activities of both the enzymes were measured in terms of $\mu$g. of phosphorus liberated per hour per $\mu$g. of protein. Phosphorus was measured by the method of Fiske and Subbarow (1925). Ornithine transcarbamylase activity was measured by the method of Burnett and Cohen (1957). Citrulline, formed as the end product, was measured by the method of Archibald (1944). Enzyme activity was expressed in terms of $\mu$g. of citrulline formed per hour per $\mu$g. of protein. Arginase activity was measured by the method of Brown and Cohen (1959). Urea, an end product of the enzyme reaction was measured by the method of Archibald (1945) and the enzyme activity was expressed in terms of $\mu$g. of urea liberated per hour per $\mu$g. of protein. Xanthine oxidase activity was measured by the method of Litwack *et al.* (1953). Enzyme activity was measured in terms of $\mu$g. of xanthine disappeared per hour per $\mu$g. of protein. Protein was estimated by the method of Lowry *et al.* (1951). Results were subjected to " t " test for small number of samples.

Enzyme activities in cell suspensions were also expressed per mg. dry weight and per cell.

### *Observation*

Fig. 1 and 2 show the phase contrast pictures of liver and tumour cell suspensions respectively. From the illustrations it may be observed that it is possible to get the cell preparations relatively free from contamination and there is no clumping of the cells. Fig. 3 shows average number of cells per mg. dry weight as well as protein content per mg. dry weight of the cell suspension from normal liver and hepatoma. It may be noted that in the tumour cell suspension, the number of cells per mg. dry weight is significantly higher than that in the parenchymal cell suspension. Similarly protein content of the tumour cell suspension is considerably higher than that of the corresponding normal group.

Table I shows the activities of G-6-Pase and FDPase in the normal liver and in tumour tissue, as well as in their corresponding cell suspensions. It is evident that the decrease in enzyme activities in the tumour cell suspension is much greater than that observed in the tumour tissue.

Table II shows the activities of ornithine transcarbamylase arginase and xanthine oxidase. Here, too, it is observed that the percentage decrease in

---

EXPLANATION OF PLATE

FIG. 1.—Phase contrast photograph of normal cells. ×1080.
FIG. 2.—Phase contrast photograph of tumour cells. ×1080.

Bhide.

activities of all the three enzymes in the tumour cell suspension is much more than that observed in the tumour tissue.

Table III indicates the activities of gluconeogenic enzymes in tumour cell suspension when measured per $\mu g$. of protein, per mg. dry weight, or per cell. It may be noted that the decrease in enzyme activities in tumour cells is highest when expressed per $\mu g$. of protein. The decrease in both the enzyme activites in tumour cells, is comparable whether it is measured per mg. dry weight or per cell.

FIG. 3.—Histogram denoting protein content and number of cells per mg. dry weight of cells.

Activities of ornithine transcarbamylase, arginase and xanthine oxidase expressed per $\mu g$. of protein, per mg. dry weight, or per cell, are shown in Table IV. Activities of all the enzymes in tumour cells are lowest when they are expressed per $\mu g$. of protein. However, on using the other two parameters the extent of decrease in enzyme activities is comparable.

TABLE I.—*Activities of Glucose-6-Phosphatase and Fructose-1-6-diphosphatase in Tumour Tissue and Cell Suspension*

| | Glucose-6-phosphatase | | Fructose-1-6-diphosphatase | |
|---|---|---|---|---|
| | Liver | Tumour | Liver | Tumour |
| Tissue | 1·25±0·13 | 0·75±0·07* (60) | 0·57±0·05 | 0·3±0·06* (50) |
| Suspension | 2·22±0·31* | 0·67±0·004* (30) | 0·49±0·02 | 0·18±0·03 (36) |

Enzyme activities are expressed in terms of μg. of phosphorus liberated per hour per μg. of protein.
Values in parenthesis are expressed as percentage of values of control group which are arbitrarily taken as 100%.
* Denotes statistically significant when $P$ value is $< 0·05$.
Experimental results represent mean of six experiments and standard error.

TABLE II.—*Activities of Ornithine Transcarbamylase, Arginase and Xanthine Oxidase in Tumour Tissue and Cell Suspension*

| Group | | Ornithine transcarb-amylase | Arginase | Xanthine oxidase |
|---|---|---|---|---|
| Tissue | Liver | 0·41±0·01 | 69·3±0·5 | 0·16±0·002 |
| | Tumour | 0·24±0·03* (60) | 40·2±0·8* (57) | 0·08±0·01* (50) |
| Cell suspension | Liver | 16·3±1·6 | 24·0±1·2 | 1·4±0·1 |
| | Tumour | 3·8±1·0* (24) | 9·0±0·4* (37) | 0·5±0·2 (36) |

Ornithine transcarbamylase activity is expressed in terms of μg. of citrulline formed per μg. of protein per hour.
Arginase activity is measured in terms of μg. of urea formed per μg. of protein per hour.
Xanthine oxidase activity is measured in terms of μg. of xanthine disappeared per μg. of protein per hour.
Values in parenthesis are expressed as percentage of values of the control group which are arbitrarily taken as 100%.
* Denotes statistically significant when $P$ value is $<0·5$.
Experimental results represent mean of six experiments with standard error.

TABLE III.—*Glucose-6-phosphatase (G-6-Pase) and Fructose-1-6-Diphosphatase (FDPase) Activities in Tumour and Parenchymal Cell Suspensions*

| | | Enzyme activity per hour | | |
|---|---|---|---|---|
| Enzyme | Group | Per μg. protein | Per mg. weight | Per cell |
| G-6-Pase | Control | 2·2±0·3 | 6·1±1·9 | 14·8±1·8 |
| | Tumour | 0·67±0·004* (31) | 3·5±1·2* (51) | 7·7±1·2* (52) |
| FDPase | Control | 0·43±0·05 | 1·1±0·3 | 2·4±1·2* |
| | Tumour | 0·2±0·04* (30) | 0·56±0·01* (50) | 1·1±0·06* (46) |

Activities of both the enzymes are expressed in terms of μg. of phosphorus liberated per hour per μg. of protein, per mg. weight and per cell.
Values in parenthesis are expressed as percentage of values of control group which are arbitrarily taken as 100%.
* Denotes statistically significant when $P$ value is $<0·05$.
Experimental results represent mean of six experiments and standard error.

TABLE IV.—*Ornithine Transcarbamylase Arginase and Xanthine Oxidase in Tumour and Parenchymal Cell Suspension*

| Enzyme | Group | Enzyme activity per hour: Per μg. protein | Per mg. weight | Per cell |
|---|---|---|---|---|
| Ornithine transcarbamylase | Control | 16·5±3·1 | 79·3±12·0 | 76·6±2·2 |
| | Tumour | 3·5±0·6* | 32±3* | 40·3±5·8* |
| | | (21) | (40) | (51) |
| Arginase | Control | 24·5±1·9 | 132·4±6·0 | 129·6±6·4 |
| | Tumour | 9·6±1·6* | 71±6·5* | 79·5±2·1* |
| | | (39) | (53) | (62) |
| Xanthine oxidase | Control | 1·4±0·1 | 7·1±1·0 | 4·1±0·4 |
| | Tumour | 0·5±0·23* | 2·8±0·29* | 1·5±0·9* |
| | | (361) | (40) | (37) |

Ornithine transcarbamylase activity is expressed in terms of μg. of citrulline formed per hour per μg. of protein, per mg. weight and per cell.

Arginase activity is measured in terms of μg. of urea formed per hour per μg. of protein, per mg. weight and per cell.

Xanthine oxidase activity is measured in terms of μg. of xanthine disappearance per hour per μg. of protein, per mg. of weight and per cell.

Values in parenthesis are expressed as percentage of values of control group which are arbitrarily taken as 100%.

* Denotes statistically significant when $P$ value is $<0\cdot05$.

Experimental results represent mean of six experiments and standard error.

### DISCUSSION

In our earlier studies on thioacetamide induced hepatomas (Bhide, 1970) we observed that activities of gluconeogenic enzymes, xanthine oxidase, ornithine transcarbamylase and arginase in liver tissue decreased progressively on thioacetamide feeding and were lowest in the tumour tissue. Use of tumour cell suspension has shown further that the magnitude of decrease in enzyme activities of tumour cells is far bigger than that observed in the composite tumour tissue. Yet it is important to state here, that so far no qualitative difference was observed in the enzymatic set up of tumour and parenchymal cells. It may be concluded from the above observations that the thioacetamide induced primary hepatoma largely resembles its parent tissue of origin and does not seem to be functionally dedifferentiated. Hence it is important to extend this search further in order to identify those minimum metabolic alterations in liver tissue which are essential for its malignant transformation. Extensive studies on minimal deviation hepatomas are being carried out with the same purpose and hope, but since these tumours are maintained in serial transplantation the factor of progression of tumour interferes. Hence the tumour cell suspension of a primary hepatoma may serve as a useful model for studies on the biochemical characterization of malignant cells. Further studies in this direction are in progress and will be reported later.

The author is grateful to Dr. K. J. Ranadive, Chief, Biology Division, for her encouragement and support in this project.

### REFERENCES

ARCHIBALD, R. M.—(1944) *J. biol. Chem.*, **156**, 121.—(1945) *J. biol. Chem.*, **157**, 507.
BHIDE, S. V.—(1970) *Br. J. Cancer*, **24**, 504.
BROWN, G. W. AND COHEN, P. P.—(1959) *J. biol. Chem.*, **234**, 1769.

Burnett, G. H. and Cohen, P. P.—(1957) *J. biol. Chem.*, **229**, 337.
Cori, G. T. and Cori, C. F.—(1952) *J. biol. Chem.*, **199**, 661.
Fiske, C. H. and Subbarow, V. J.—(1925) *J. biol. Chem.*, **66**, 375.
Gothoskar, S. V., Talwalkar, G. V. and Bhide, S. V.—(1970) *Br. J. Cancer*, **24**, 498.
Jacob, S. T. and Bhargawa, P. M.—(1962) *Expl Cell Res.*, **27**, 453.
Litwack, G., Bothwell, J. W., Williams, J. N. and Elvehjem, C. A.—(1953) *J. biol. Chem.*, **200**, 303.
Lowry, O. H., Rosebrough, N. J., Farr, A. L. and Randall, R. J.—(1951) *J. biol. Chem.*, **193**, 265.
Pogell, B. M. and McGilvery, R. W.—(1954) *J. biol. Chem.*, **208**, 149.
Ranadive, K. J.—(1957) *Coll. Pap. Lab. Anim. Bur.*, **6**, 37.